# Endovascular embolization of canine hepatic arteriovenous malformations using precipitating hydrophobic injectable liquid (PHIL) liquid embolic agent: a proof of concept study

**DOI:** 10.1186/s42155-019-0070-4

**Published:** 2019-08-01

**Authors:** Stewart D. Ryan, Anjali Nambiar, Julian Maingard, Hong Kuan Kok, Robert B. S. Turner, Duncan Mark Brooks, Hamed Asadi

**Affiliations:** 1 TRACTS, UVet Hospital, Melbourne Veterinary School, Faculty of Veterinary and Agricultural Sciences, The University of Melbourne, Melbourne, Australia; 2 School of Medicine and Dentistry, James Cook University, Townsville, Australia; 3 Interventional Radiology Service, Department of Radiology, Austin Hospital, 250 Princes Highway,Werribee, Melbourne, Victoria 3030 Australia; 4 Interventional Neuroradiology Service, Radiology Department, Austin Hospital, Melbourne, Australia; 5 School of Medicine, Faculty of Health, Deakin University, Waurn Ponds, Australia; 6 Interventional Neuroradiology Unit, Monash Health, Melbourne, Australia; 7 Interventional Radiology Service, Northern Hospital Radiology, Melbourne, Australia

**Keywords:** Hepatic arteriovenous malformation, PHIL, Liquid embolic, Scepter XC

## Abstract

**Background:**

Hepatic arteriovenous malformations (HAVMs) are rare congenital lesions consisting of multiple high-pressure arteries feeding into low-pressure veins via a central nidus. Massive haemorrhage, portal hypertension and hepatic insufficiency can ensue. Endovascular embolization is increasingly a first line treatment method although there is no general consensus or guidelines on the most effective embolic agent or approach. We describe the novel treatment of two dogs with congenital hepatic AVMs using a modified version of the ‘pressure cooker’ technique often utilised in neurointervention with the DMSO-based PHIL embolic agent delivered via the DMSO compatible Scepter-XC dual lumen balloon catheter.

**Case presentation:**

Two paediatric dogs were diagnosed with hepatic AVMs. Both dogs presented with ascites and abnormal liver function tests. CT angiograms revealed hepatic arterio-portal malformations arising from an enlarged celiac artery. Selective catheterisation of the artery supplying the AVM was achieved via a femoral artery approach. A Scepter XC dual-lumen compliant balloon microcatheter and Traxcess 0.014 guidewire combination was advanced to the nidus via through the 5Fr guide catheter towards the nidus. Inflation of the balloon occluded arterial inflow and PHIL was injected under continuous fluoroscopic screening until the PHIL embolic agent penetrated into the draining portal vein beyond the nidus.

In patient 1, normal portal venous waveform was restored with reversal of severe hepatic insufficiency. Whilst there was initial improvement post-operatively in patient 2 with normalisation of portal vein pressures and flow, opening of collateral nidus vessels re-established the high-pressure communication, and euthanasia was elected by the owner.

**Conclusions:**

The ‘pressure cooker’ technique is a safe and efficacious approach to the treatment of canine HAVMs. The novel use of PHIL and the Scepter XC balloon catheter has several advantages over conventional endovascular approaches. Translational application to human paediatric interventions for similar conditions where embolic and contrast agent volume constraints are similar can be considered.

## Introduction

Hepatic arteriovenous malformations (HAVM) are characterised by high pressure arteries forming abnormal connections with either low pressure hepatic or portal veins in a central nidus, bypassing the normal intervening capillary bed. (Whitehead & Dean, 2013) Abnormal communications between the hepatic artery and vein manifest in high output cardiac failure, whilst arterio-portal malformations cause portal hypertension, ascites and hepatic insufficiency. (Caselitz et al., 1998) Endovascular embolization is increasingly the first line in the treatment of AVMs in humans and dogs due to its minimally invasive nature and reduced surgical-related morbidity and mortality. (Bolus et al., 2014; Chanoit et al., 2007) More recently, the use of Dimethyl sulfoxide (DMSO) liquid based embolic agents, Onyx^1^ and PHIL (Precipitating Hydrophobic Injectable Liquid),^2^ has shown increasing efficacy and long term occlusion rates in humans and dogs due to superior deliverability and nidal occlusion. (Vollherbst et al., 2017) Here we report the endovascular treatment of two canine HAVMs using the DMSO/EVOH based PHIL liquid embolic agent achieved with a modified version of the “pressure cooker” embolization technique used extensively in neurovascular intervention for cerebral AVMs. (Vollherbst et al., 2017; Chapot et al., 2014) We also describe the use of the Scepter XC DMSO-compatible compliant balloon microcatheter^3^ as a novel use in the peripheral circulation rather than the brain circulation for treatment of HAVMs.

## Materials and methods

This was a retrospective case series of two dogs with congenital HAVM treated with arterial embolization using PHIL.

### Patient 1

A 6-month-old male-neutered Dachshund presented with weight-loss, vomiting, loss of appetite, diarrhoea, and intermittent acute per rectal bleeding over a 3-month period. Initial diagnostic workup was directed at the suspected diagnosis of the more common condition of portosystemic shunt seen in dogs. Medical treatment was initiated at the primary care facility with Metronidazole (50 mg PO BID) and Lactulose (1 ml PO TID).

**Fig. 1:**
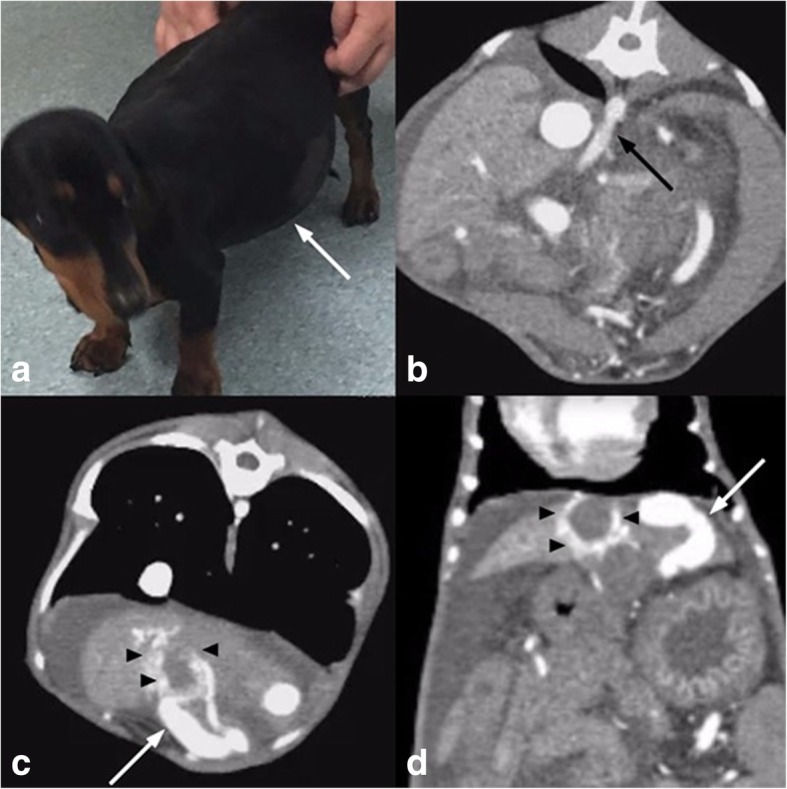
Patient 1 presenting with **a**) tense ascites (arrow) and biochemical hepatic dysfunction. **b**) Preoperative CT angiogram demonstrating an enlarged celiac artery (arrow) and extensive ascites. **c**) and **d**) demonstrate an AVM nidus surrounding the gallbladder fossa (arrow heads) with an enlarged and arterialised draining portal vein branch (arrow)

At presentation the dog had tense ascites, (Fig. 1a). abnormal liver function tests, a prolonged activated partial thromboplastin time (aPTT) and hypoproteinemia (Table 1). Computed tomographic angiography (CTA) was performed and revealed a hepatic arterio-portal malformation arising from an enlarged celiac artery, a nidus centred on the gallbladder and arterialisation of an aberrant left intra-hepatic portal vein. (Fig. 1b, c and d) Multiple varices were also present, with moderate ascites, splenomegaly and moderate bilateral renomegaly.

**Table 1:** Selected biochemical and haematology parameters at various pre and post procedure timepoints for Dog 1 and Dog 2. Bold values represent values outside the normal canine reference ranges

Parameter	Dog 1				Dog 2		Normal canine REF range (units)
	Pre Op	1 month Post Op	6 months Post Op	18 months Post Op	Pre Op	1 week Post Op	
UREA	**2.2**	**3.3**	**2.3**	**2.3**	**1.1**	**1.0**	3.0–8.7 (mmol/L)
CREA	**24**	**28**	**21**	**18**	**24**	N/A	40–140 (μmol/L)
ALT	**385**	**481**	**320**	**131**	**177**	**403**	3–83 (U/L)
ALKP	**641**	**409**	**255**	151	**272**	**371**	0–170 (U/L)
T BILI	0	1	N/A	1	6	N/A	0–20 (μmol/L)
TP	59	57	**49**	57	**50**	**42**	51–72 (g/L)
ALB	**30**	33	**24**	**28**	**22**	**29**	31–44 (g/L)
GLOB	29	24	25	29	28	**13**	14–37 (g/L)
GLUC	5.1	4.8	4.1	4.4	5.3	N/A	3.4–7.4 (mmol/L)
CHOL		**3.3**		**2.8**	**3.1**	5.3	3.9–7.8 (mmol/L)
Pre bile acids	**107**	**168**	N/A	**366**	N/A	N/A	0–15 (mmol/L)
Post bile acids	**204**	N/A	N/A	**270**	N/A	N/A	0–15 (mmol/L)
^1^pcv	0.18	0.39	0.42	0.46	0.28	0.31	0.37–0.55 (L/L)
WBC	19.6	16.1	14.2	11.7	13.8	12.5	6.0–17.0 (× 10^9^/L)
PLT	376	468	282	**72**	336	210	200–500 (× 10^9^/L)
PT	8.0						6.9–8.8 (secs)
APTT	**18.0**						13.1–17.2 (secs)

Entries in bold font indicate values outside the normal canine reference range

**Fig. 2:**
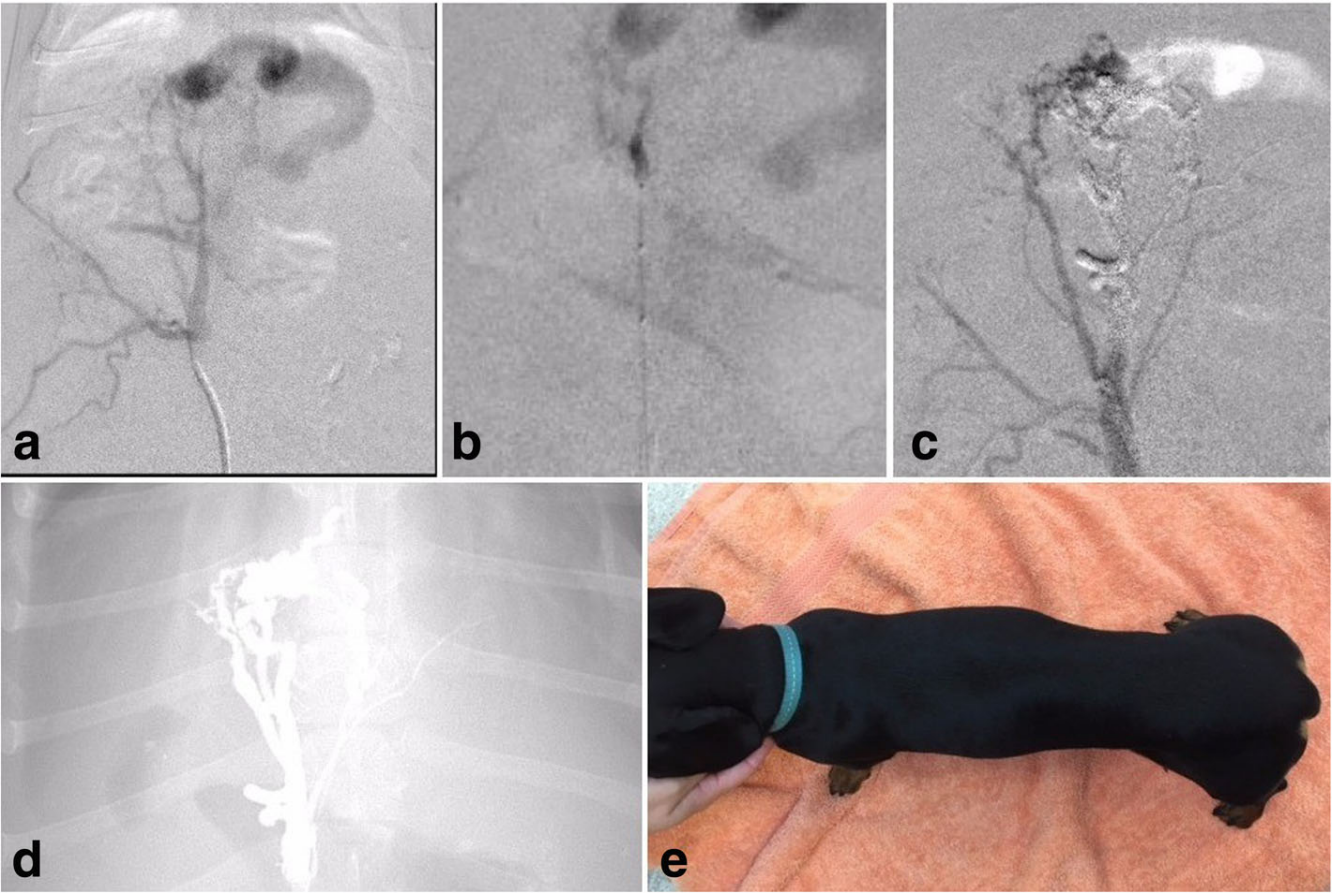
Embolization imaging for patient 1. **a**) Preliminary DSA demonstrating the hepatic AVM nidus within the gallbladder fossa with a large draining arterialised left portal vein branch, **b**) to **d**) PHIL embolization via the Scepter XC dual lumen compliant balloon microcatheter with resultant embolic cast. **e**) Three days post embolization with resolution of tense ascites

Under general anaesthesia, and following percutaneous drainage of a large volume of clear ascites, vascular access was gained via the femoral artery with a 4Fr micropuncture kit.^4^ Pre-operative abdominal ultrasound examination showed a dilated portal vein and tributaries with biphasic pulsatile retrograde flow (peak velocity ~ 50 cm/s). The velocities within the left hepatic branch of the portal vein were pulsatile and retrograde with peak velocity ~ 120 cm/s just distal to the truncation. A 0.035″ hydrophilic guide wire surrounded by a 5Fr vascular sheath and a Chaperon guiding catheter^5^, which was composed of a coaxial system combining of outer 5Fr and inner 4Fr, was used to selectively catheterise the celiac artery under fluoroscopic guidance (Fig. 2a). The inner 4Fr catheter was removed and the 5Fr outer catheter remained in situ. Heparinised saline (1000 IU in 500 ml) was infused via a Tuohy-Borst adapter^6^ during the procedure. A Scepter XC dual-lumen compliant balloon microcatheter and Traxcess 0.014 guidewire^7^ combination was advanced through the 5Fr guide catheter towards the nidus. Inflation of the balloon occluded arterial inflow (Fig. 2b). After flushing with DMSO, PHIL was injected through the Scepter XC balloon microcatheter under continuous fluoroscopic screening to monitor for complete filling of the nidus and to detect any potential reflux into the parent artery or non-target embolization. Injection was terminated when the PHIL embolic agent penetrated in to the draining left portal vein beyond the nidus (Fig. 2c). The occlusion balloon was the deflated and retracted slowly to ensure that the PHIL embolic material had not adhered to the microcatheter and had not entrapped it. Subsequent digital subtraction angiography (DSA) revealed no remaining flow through the arterio-portal communication (Fig. 2d), confirming that the HAVM was successfully treated in entirety. The femoral artery was ligated, and a subcutaneous simple cutaneous suture closure was performed. Post-surgical ultrasound examination revealed non-pulsatile, retrograde blood flow within the portal vein (12 cm/s) and the dilated left portal vein (10 cm/s).

### Patient 2

**Fig. 3:**
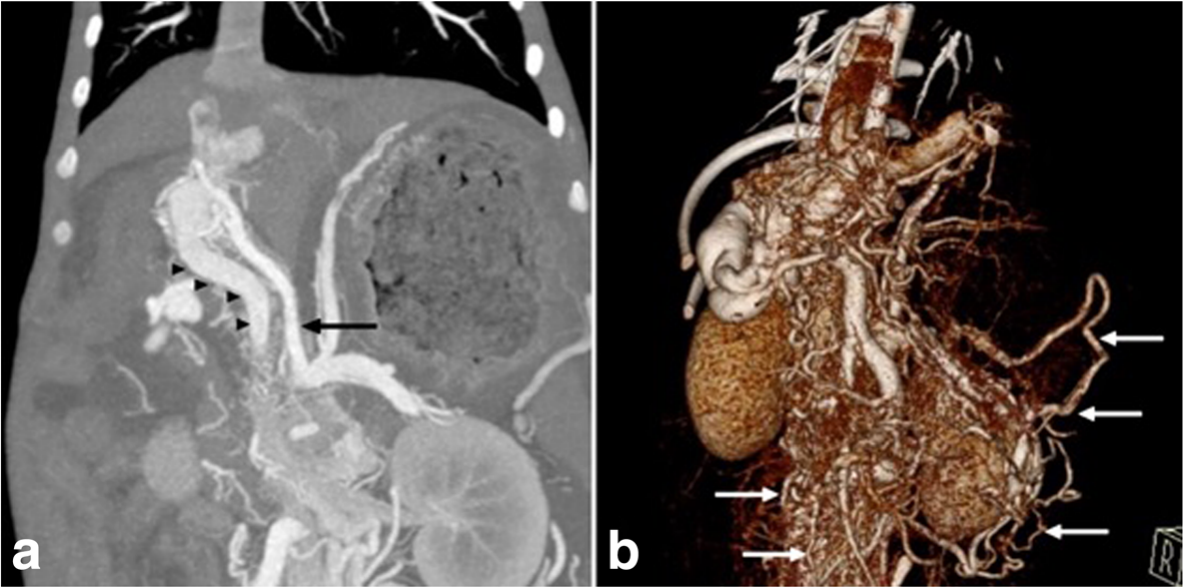
Preprocedural imaging in patient 2. **a**) CT angiography demonstrates a large hepatic AVM with arterial inflow arising from an enlarged celiac artery (black arrow) with early shunting into an enlarged and arterialised portal vein (arrow heads). **b**) 3D volumetric reconstruction shows innumerable portosystemic collaterals (white arrows)

**Fig. 4:**
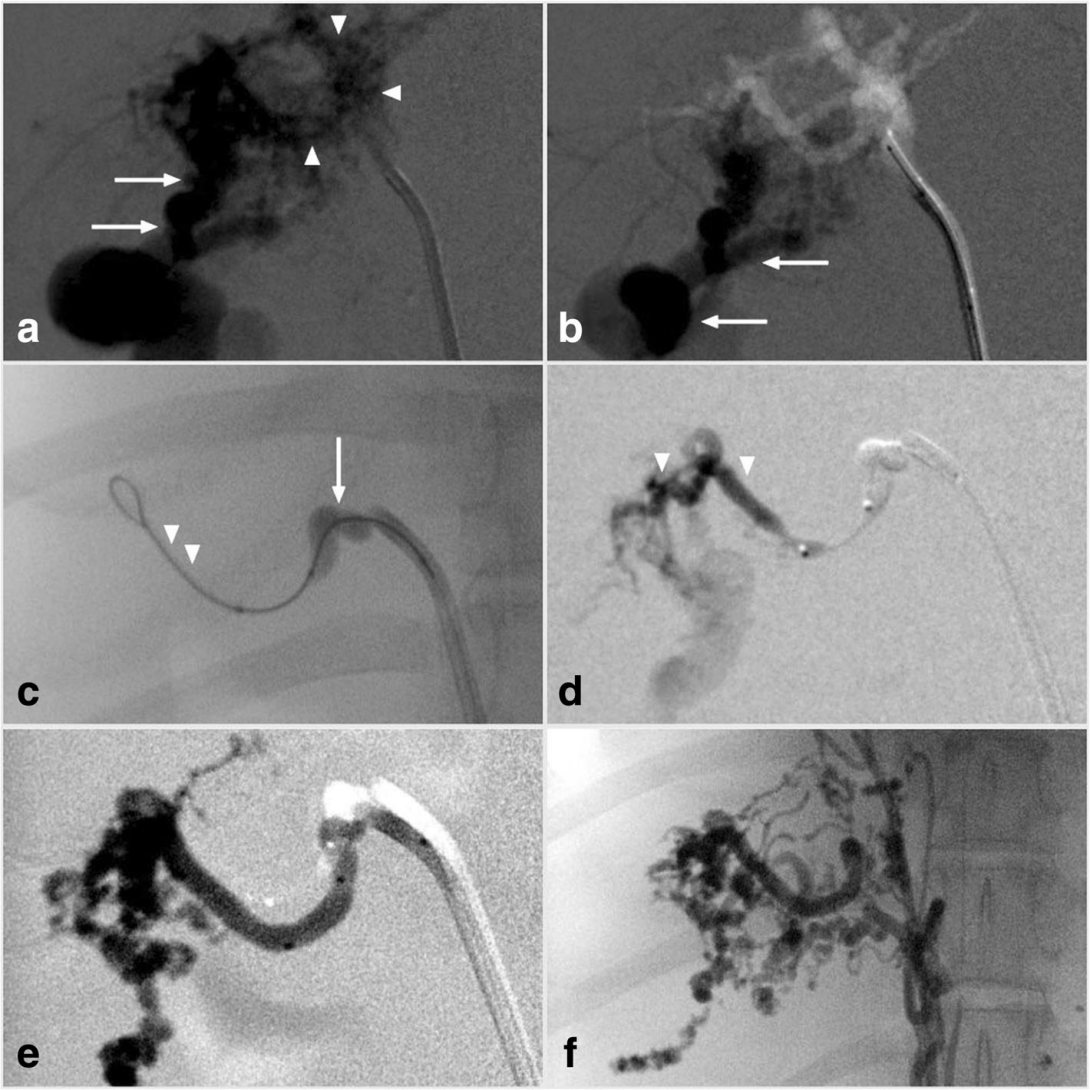
Hepatic AVM embolization in patient 2. **a**) and **b**) Enlarged and tortuous hepatic and celiac arteries supplied the nidus (arrowheads) with early portovenous shunting (arrow). **c**) The Scepter XC dual lumen balloon microcatheter (arrow) and 0.014″ guidewire (arrowheads) combination were advanced into the main hepatic arterial branch. Balloon inflation occluding most of the arterial inflow. **d**) and **e**) PHIL liquid embolic (arrowheads) was injected into the AVM nidus until stagnation was achieved. **f**) Completion DSA showed no residual arterial inflow or portovenous shunting

A 23-week-old, male Labrador dog presented with weight loss and ascites. The clinical symptoms and serum biochemistry were consistent with portosystemic shunting (Table 1). Abdominal ultrasonography, CTA and DSA demonstrated a large HAVM similar to patient 1, with nidus arterialisation of a right intra-hepatic portal vein (Fig. 3a, 4a and b) and multiple acquired shunts and portal thrombosis (Fig. 3b). Portal hypertension with pulsatile hepatofugal with flow velocities exceeding 70 cm/s was documented. The celiac artery diameter was 1.5 times the cranial mesenteric artery diameter.

The procedural setup and approach was similar to patient 1. The Scepter XC microcatheter and Traxcess 0.014″ guide wire combination was navigated to the arterial feeder. The balloon of the Scepter XC microcatheter was inflated within the main hepatic arterial branch supplying the nidus occluding most of the arterial inflow (Fig. 4c). PHIL liquid embolic was injected to a similar angiographic endpoint as in patient 1 until stagnation of flow was achieved (Fig. 4d and e). Completion DSA showed no residual arterial inflow or portovenous shunting. (Fig. 4f).

## Results

### Patient 1

**Fig. 5:**
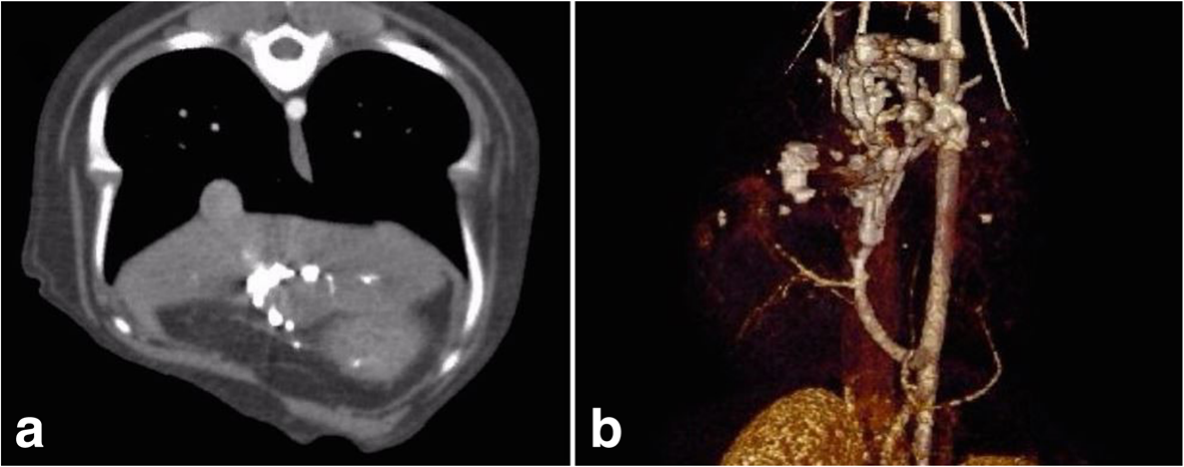
Post procedural CTA in Patient 1**a**) Dense PHIL cast within the gallbladder fossa without residual arterial flow. **b**) 3D volumetric reconstruction shows the PHIL embolic cast without evidence of recurrent or residual AVM

Repeat abdominal ultrasound post-embolization showed reduced hepatofugal velocity in the portal vein and smaller portal vein diameter. The ascites resolved within 3 postoperative days (Fig. 2e). Six-week follow-up ultrasonography showed that the portal vein at the level of the splenic vein was no longer dilated (3.2 mm diameter) relative to the pre-operative ultrasound (~ 7 mm diameter) and laminar, non-pulsatile hepatopetal flow was present with peak flow velocity of ~ 4 cm/s). CTA revealed subjectively increased hepatic volume without AVM recurrence (Fig. 5a and b).

Telephone conversation with the owner 6 months after embolization reported no delayed complications, normal activity and weight gain on a diet without any medications.

Re-examination, including clinical examination, blood tests and imaging with abdominal ultrasonography and contrast CT scan was done at 18 months after the procedure. No ascites was present and the liver remained small with normal margins and echogenicity/echotexture. The portal vein could be followed from the cranial mesenteric vein and splenic vein into the left and right intra-hepatic branches. Laminar, non-pulsatile cranial flow was present with peak velocity of current 7.6 cm/s compared to previous value of 4 cm/s. The dilated left portal vein and aberrant vessel could no longer be identified. Heterogeneously hyperechoic material that displaces the wall of the gallbladder was present. The previously described nidus of vessels around the gallbladder were no longer identified.

### Patient 2

Immediate postoperative ultrasonography revealed reduced hepatofugal velocity (~ 10 cm/s) in the portal vein. However, his condition deteriorated over the following days with re-accumulation of ascitic fluid, vomiting, weight loss and pancreatitis. Abdominal ultrasonography 1 week after the embolization procedure revealed re-arterialisation with increased portal vein hepatofugal flow that peaks at 45 cm/s. The patient was euthanized at the owner’s request based on morbidity and financial considerations.

## Discussion

This case series proof of concept study demonstrated that the use of PHIL as an embolization agent using the balloon occlusion microcatheter pressure cooker technique via the Scepter XC was technically feasible and was safe and efficacious in the treatment of the canine HAVMs.

In humans, liquid embolic agents are the preferred treatment for brain AVMs, with N-butyl cyanoacrylate and Onyx being used most frequently. (Vollherbst et al., 2017; Leyon et al., 2017) Onyx has also been successfully used for the treatment of peripheral and hepatic AVMs in dogs, highlighting that liquid embolic agents that are successful in human brain AVMs have similar benefits and outcomes in canine species. (Chanoit et al., 2007; Vollherbst et al., 2017; Culp et al., 2014)

New embolic agents have been investigated to improve the efficacy of AVM obliteration, intraprocedural handling and fluoroscopic visibility. An ideal embolic agent should provide permanent embolization and low chance of recanalization. The precipitation time of the embolic agent should be short and should have no toxigenic effects on the tissues. (Vollherbst et al., 2017) PHIL is a relatively new liquid embolic agent consisting of a non-adhesive copolymer (polylactide- co-glycolide and polyhydroxyethylmethacrylate) which is dissolved in dimethyl sulfoxide (DMSO), with triiodophenol as the iodine component. (Vollherbst et al., 2017) Studies where PHIL has been used in the treatment of brain AVMs in humans have shown promising results, with PHIL offering numerous advantages compared to other embolic agents. PHIL’s relative ease of use is highlighted by faster plug formation, good forward flow and less CT and MRI artefacts during follow up imaging compared to tantalum-containing materials such as Onyx. (Kocer et al., 2016). PHIL also takes up iodinated contrast to provide radio-opacity, leading to an increased capacity for target embolization. PHIL therefore overcomes the limitations of other liquid embolic materials, where there are more imaging artefacts and forward flow is difficult. (Leyon et al., 2017) PHIL is available in ready to use syringes and has a precipitation time of approximately 3 min, compared to Onyx which requires up to 20 min of preparation time and has a precipitation time of 5 min. Furthermore, PHIL is available in three concentrations: PHIL 25, 30 and 35, each increasing in viscosity and concentration, with the least viscous formulation being used for deeper penetration and more effective filling of the AVM nidus. (Vollherbst et al., 2017) The use of PHIL as an embolic agent has been shown to require less product to achieve the same occlusive effect as other embolic agents. (Vollherbst et al., 2017) Both PHIL and Onyx exhibit dose-related angiotoxic effects, however angionecrosis was shown to be absent in PHIL-filled vessels during the treatment of human brain AVMs, thus making it a potentially safer agent for a paediatric population. However, it has been shown that waiting times between injections had a considerable effect on the degree of embolization using PHIL, which may increase the likelihood of technical errors such as subtotal filling of the AVM due to premature embolization of the proximal AVM or distal off-target embolization during the procedure. (Vollherbst et al., 2017) As PHIL shows promise as a safe and effective embolic agent, further trials may be necessary to establish its use in the treatment of HAVMs in dogs and humans.

Although HAVMs in dogs and humans differ in terms of relative prevalence and the vessels involved, the overarching similarities in the vascular malformation allows translational experience for imaging and treatment modalities between the two species. HAVMs in humans and dogs are angiographically similar, with a network of arteries connecting to veins via a central nidus, suggesting that embolization techniques are likely to have similar results in both species. In dogs, the communication is between the hepatic artery and portal vein, whereas in humans, communication is typically between the hepatic artery and hepatic vein. (Raj, 2015) As PHIL has been successfully used in the treatment of cerebral and spinal arteriovenous fistulas in humans, its use may be extended to treating hepatic AVMs in the paediatric population. There are a lack of trials exploring the use of PHIL in the human paediatric population, with patients treated with PHIL for cerebral AVMs having a mean age of 58. (Leyon et al., 2017) However, given the dogs in this study are young and of small size, PHIL may potentially be a safe and effective agent for the endovascular embolization of HAVMs in the paediatric population, although the long-term outcomes of treatment with PHIL are yet to be explored.

The use of the Scepter XC DMSO-compatible dual lumen compliant balloon catheter in the peripheral circulation for the treatment of HAVMs is a novel application as it has previously only been described for use intracranial and cervical AVMs in humans. (Jagadeesan et al., 2013) Whilst other single-lumen balloon catheters require a balloon specific 0.010″ guide wire, the Scepter XC balloon catheter is compatible with a 0.014″ guide wire, which allows for easier navigation through tortuous arteries with a greater degree of steerability and trackability, allowing for better repositioning of the balloon catheter. The Scepter XC balloon catheter also has a soft distal tip that allows for steam shaping, allowing tortuous arteries to be tracked with greater precision whilst inhibiting blood reflux back into the balloon. (Rho et al., 2013) Because reflux of the embolic agent is avoided with the use of Scepter XC, the embolization of the AVM is improved and complications such as proximal branch occlusion or entrapment of the catheter is avoided. (Kin & Kong, 2017) Additionally, the use of Scepter XC does not show problems associated with the repeated inflation and deflation of the balloon. (Rho et al., 2013) Although a proximal Onyx plug is essential for many AVM embolizations, it is not required when the Scepter XC balloon is used which increases the efficacy of AVM occlusion allowing for high-pressure embolic injection in the “pressure cooker” technique. (Chapot et al., 2014) The principle of the pressure cooker technique is to create a plug to avoid backflow reflux and enable a more comprehensive, forceful and continuous injection of embolic agent through the AVM nidus vessels. Fluoroscopy and procedural times have also reported to be lower for embolization procedures using Scepter XC compared to conventional balloon catheters. (Jagadeesan et al., 2013)

## Conclusion

This case series proof of concept study demonstrated that the technical feasibility of PHIL as an embolization agent using the balloon occlusion microcatheter pressure cooker technique via the Scepter XC was safe and efficacious in the treatment of the canine HAVMs. The use of Scepter XC balloon as a novel DMSO compatible balloon catheter provided easier navigation and greater controllability during HAVM embolization. The procedural success and clinical outcomes in these dogs has important potential translational implications for treatment of similar conditions in a paediatric population where the embolic and contrast agent volume constraints are similar.

## Data Availability

The datasets (medical records and diagnostic imaging) used and/or analysed during the current study are available from the corresponding author on reasonable request.

## Abbreviations

AVM: Arteriovenous malformation
DMSO: Dimethyl-sulfoxide
EVOH: Ethylene-vinyl alcohol copolymer
HAVM: Hepatic arteriovenous malformation
PHIL: Precipitating hydrophobic injectable liquid

## Footnotes

1 Micro Therapeutics, Inc., Irvine, CA

2 Microvention, CA, USA

3 Microvention, CA, USA

4 Cook Medical, IN, USA

5 Chaperon; Terumo Microvention, Tokyo, Japan

6 Cook Medical, IN, USA)

7 Microvention, CA, USA
